# The progestin revolution 2: progestins are now a dominant player in the tight interlink between contraceptive protection and bleeding control—plus more

**DOI:** 10.1186/s40834-023-00249-5

**Published:** 2023-10-09

**Authors:** Donna Shoupe

**Affiliations:** Keck School of Medicine, University of Southern California, Los Angeles, CA USA

## Abstract

The interlink between bleeding control and contraceptive development has always been an important factor. But after many years of advances in contraceptive technology, this interplay has resulted in development of safer and better contraceptive methods that often offer significantly less bleeding for women with both normal bleeding patterns as well as in those suffering from heavy menstrual bleeding (HMB). Recognition of the success of progestin-only methods, such as the hormonal IUDs, progestin dominant oral contraceptives, and the high dose progestin-only pill in substantially decreasing and controlling menstrual bleeding has led the way. This recognition also led to the development of many [non-contraceptive] protocols to stop acute heavy bleeding as well as manage long-term bleeding [using contraceptive methods as well; as non-contraceptive methods].

But even better, there is a new PLUS. The distinct benefit and risk profiles of the many different progestins now available are intentionally being used either in combination contraceptive pills [COCPs] or alone, to add additional benefits, to decrease side effects and risks, and increase effectiveness and bleeding control.

## A very brief history of the development and advances in contraceptive products – improvements in safety, efficacy and bleeding control

The first two FDA approved contraceptive methods, Enovid pill and depo-medroxyprogesterone were first approved for bleeding control or cancer treatments [1, 2] In June 1960, Enovid [containing 9.85 mg norethynodrel and 150 μg mestranol], was approved as the US’s first oral contraceptive pill For over 30 years depo-medroxyprogesterone had been widely used for bleeding control before it was approved as a contraceptive by the FDA in 1992.

Early progress in contraceptive technology resulted in dramatic reductions in both the estrogen and progestin components making the pill safer and less side effects. Lowering the estrogen dose in oral contraceptives lowered the risk of increases in clotting factors and thromboembolic risk As the dose of various progestins needed to suppress ovulation became better understood, the trend of lowering the estrogen dose and increasing the progestin dose accelerated [3]. The other advantages of a lower estrogen dose and increased progestin dose was a decrease in the amount of bleeding and improvements in bleeding control.

## There was a big change in the recognition of the efficacy of high dose progestin for bleeding control and suppression of ovulation

For many years, it was thought that high progestin-only methods would cause “endometrial atrophy and increased breakthrough bleeding.” It was a general opinion that “there had to be high dose estrogen to prevent endometrial atrophy that would cause abnormal break-through bleeding”. Most of the protocols used high dose IV estrogen or multiple COCPs/day to stop acute bleeding. This opinion began to change as there was a gradual appreciation of the bleeding control seen with the progestin containing IUDs (delivering a very high dose of progestin to the endometrium), the high dose progestin-only 24–4 pill, and the new higher progestin lower estrogen COCPs [4].

The progestin revolution had begun. The revolution was spurred forward by the publication of multiple articles reporting high success rates with use of progestins [without added estrogen] for controlling and stopped both acute and chronic bleeding problems.

*Adding more progestin*, not estrogen, to treat abnormal bleeding started to become the more reliable protocol. The progestin IUD as well as the menopausal model of no bleeding despite a progestin dominant or atrophic functional endometrial lining added further evidence to this argument [4]. Progestin-only protocols also had less side effects and better tolerability.

Contraceptive technology of today continues to introduce more and more effective products that significantly reduce or eliminate monthly bleeding. The estrogen component of the pill was reduced from150 ug to 80 μg to 50 μg, and then to 30–35 μg, then 25 μg, and then 20 μg. In 2010, a 10 μg estrogen combination pill with norethindrone acetate with a 24–4 pill protocol was introduced. These changes resulted in better bleeding control and decreased overall bleeding. Other methods used to improve efficacy, safety and bleeding control is shown in (Table 1).

**Table 1:** Changes in oral contraceptives done to increase efficacy, improve safety, get better control of bleeding and obtain decreased bleeding

Introduction of biphasic and triphasic COCP regimens
Continuous COCP regimens
Change from 21–7 to 24–4 COCP regimens [less pill free days, less days of unopposed estrogen]
Addition of 2 days of estrogen during “pill free period” to control bleeding 24–2-2 regimen
Lower estrogen dose
Increased progestin dose
Use of various new progestins
Various other COCP regimens
High dose progestin-only pills

## Many advantages of less menstrual bleeding for women

Many women in modern developed countries choose to have fewer children and thereby experience significantly more menstrual cycles than women in the past. Women in underdeveloped countries tend to have more children and may only experience 40 menstrual bleeding in a lifetime while many women in developed countries can experience more than 400 menstrual periods. Many women have important roles at the home and in the workplace where irregular bleeding, menstrual cramps or heavy menstruation can cause significant socio-economic problems [5, 6]. Use of modern contraceptives methods, now designed to reduce menstrual bleeding in addition to contraceptive protection, can give women “I got my life back”.

- Abnormal menstrual bleeding affects 20–30% of premenopausal women and more than 800,000 women look for treatment annually in the UK [5].
- Abnormal uterine bleeding (AUB) in reproductive-age women (defined as abnormal in duration, quantity, or timing) occurs in one-third of all women throughout their lifetime that often impair their daily activities.
  - A US study reported financial losses of > $2000 per patient each year due to work absence and home management costs [5].
- The reported prevalence of dysmenorrhea in women is 16–91% while severe pain is reported in 2–29% of women [6].
- The perimenopause is associated with the “roller-coaster” of hormones that may cause heavier, more frequent, and less predictable bleeding.
- Women may spend as long as 39 years of monthly bleeding episodes.
- The average monthly blood loss is 30–40 ml but abnormal bleeding called menorrhagia is by definition > 60 ml.
  - Women with heavy bleeding may use large pads or towels, may need to change pads hourly, and may bleed through clothes or bedding.
  - Heavy bleeding can result is severe anemia, need of blood transfusions, emergency surgery, and may in anovulatory women be associated with endometrial hyperplasia or cancer.

## Approved protocols and popular published protocols to control and suppress abnormal uterine bleeding

Many progestin-only protocols are as effective in stopping acute bleeding as protocols using high dose estrogen plus progestin pill or estradiol alone, and generally associated with fewer side effects [7, 8]. Progestin-only regimens along with the combination protocols are now included as first line treatment options as listed below.

After the bleeding has been controlled, multiple treatment options are available for long-term management of chronic AUB. Effective medical therapies include the levonorgestrel intrauterine system, COCPs (monthly or extended cycles), [preferably high dose] POPs, progestin therapy (oral or intramuscular), tranexamic acid, and nonsteroidal anti-inflammatory drugs 7. If a patient is receiving IV conjugated equine estrogen, the health care provider should promptly add progestin containing regimen.

- ACOG Committee Opinion recommends multiple therapies to manage acute AUB [9] in adults and reaffirmed for adolescents [10]
  - IV conjugated equine estrogen [25 mg every 4–6 h × 24 h]
  - Monophasic OCPs with 30–50 μg ethinyl estradiol [every 6–8 h until cessation of bleeding]
  - Tranexamic acid [1.3 g oral or 10 mg/kg IV 3 × daily for 5 days]
  - Medroxyprogesterone acetate [20 mg 3 x/day × 7 days]
- A European Consensus group offered 4 oral options for hormonal treatment of acute bleeding in women without underlying bleeding disorders [11] Each treatment option had a recommended taper protocol.
  - Birth control pills with either 30 mcg or 50 mcg of ethinyl estradiol (EE) in combination with any progestin to be taken every 6 h until bleeding stops (with a re-evaluation at 48 h)
  - Norethindrone acetate 5 mg–10 mg every 4 h
  - MPA 10 mg every 4 h (up to 80 mg per day)

*For immediate control of heavy bleeding in women*, many publications report the effectiveness of protocols using MPA acetate oral with the depo-medroxyprogesterone injection. Patients should be hemodynamically stable but could have anemia. This is an option “when you really need to stop the bleeding” [12–14].

- Injection of depo-MPA 150 mg intramuscularly combined with 3 days of oral MPA acetate 20 mg 3 times/daily × 3 days is an effective outpatient therapy for acute abnormal uterine bleeding [15].
  - An effective progestin-only option for hemodynamically stable women with very heavy, acute bleeding and anemia [15] 48 women, 19–53 years, non-randomized study
  - Baseline hemoglobin = 10.9 g/100 mL
  - All 48 women stopped bleeding within 5 days, Mean time to bleeding cessation was 2.6 days.
  - Side effects were infrequent and patient satisfaction was high.

## The progestin revolution PLUS: The differences in progestins is now being recognized and utilized

There have been four generations of progestins. Each generation has different qualities that have additional benefits and some added risks.

### First generation progestins

Norethindrone, norethindrone acetate, and ethynodiol diacetate

#### Norethindrone

**Fig. 1:**
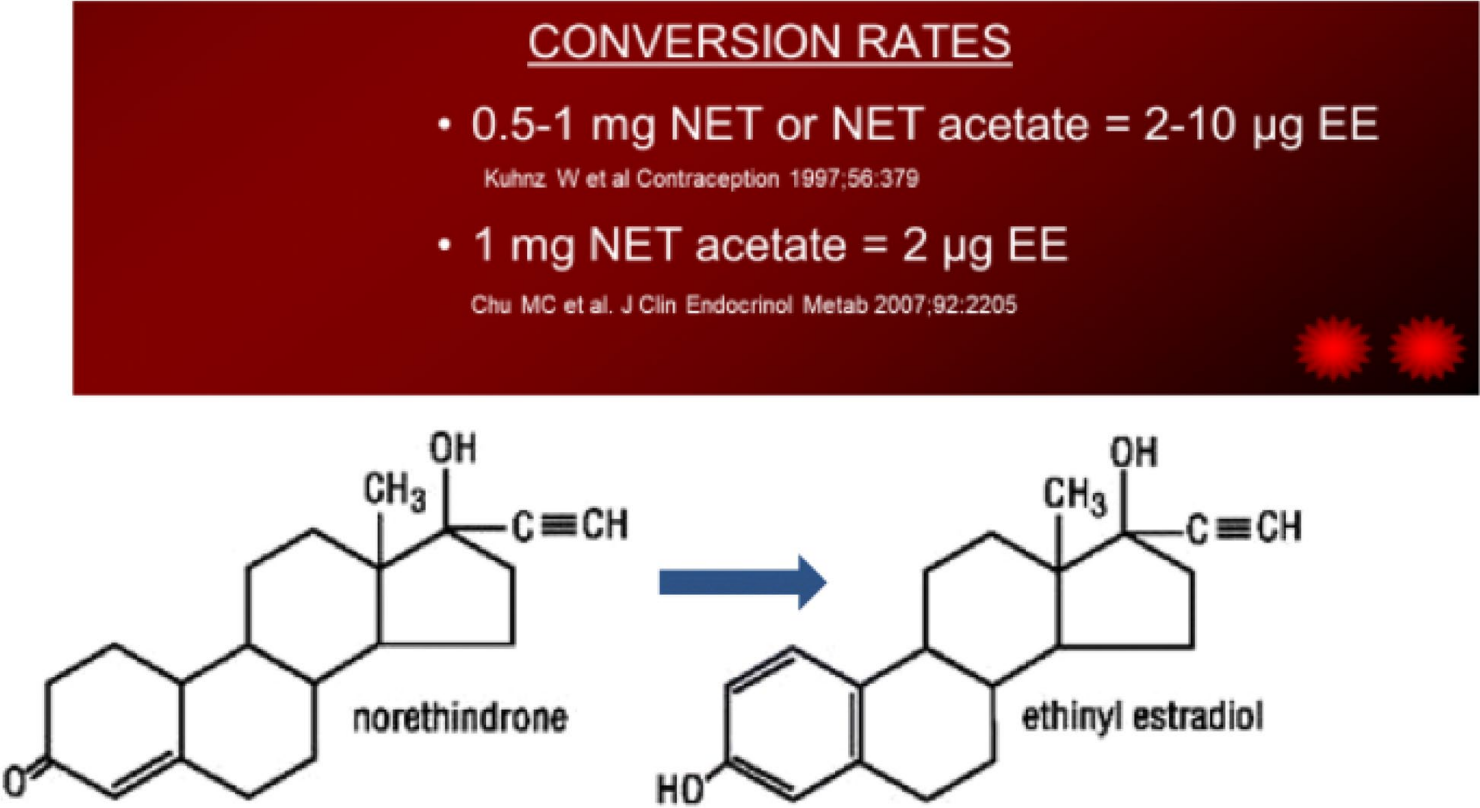
Coversion rates of NET and NET acetate to ethinyl estradiol

- Low progestational activity
- Years of data on safety
- Less androgenic than second-generation progestins but more than third and fourth generation progestins
  - ◦ Advantage: the androgenic activity in this progestin is beneficial when used in a combined oral contraceptive as it antagonizes estrogen’s stimulation of hepatic proteins [most importantly has some effect in reducing the increase in clotting factors caused by the estrogen in the COCP]
  - ◦ Disadvantage: Low antiandrogenic activity [depends on estrogen in COCPs for reducing acne]
- Converted to estrogen: conversion rates of norethindrone and norethindrone acetate to ethinyl estradiol via aromatase [16, 17] see Fig. 1

#### Norethindrone acetate [very similar to norethindrone]

- Low progestational activity
- Years of data on safety
- Less androgenic than second-generation progestins but more than third and fourth generation progestins
  - ◦ Advantage: the androgenic activity in this progestin is beneficial when used in a combined oral contraceptive as it antagonizes estrogen’s stimulation of hepatic proteins [most importantly has some effect in reducing the increase in clotting factors caused by the estrogen in the COCP]
  - ◦ Disadvantage: No antiandrogenic activity [depends on estrogen in COCPs for reducing acne]
- Converted to estrogen: conversion rates of norethindrone and norethindrone acetate to ethinyl estradiol via aromatase [16, 17] see Fig. 1
- While NET acetate 5 mg daily has not been shown to have contraceptive action and should not be used in that way, NET acetate can be used as a hormone replacement product in younger women with premature menopause or oophorectomy when estrogen is needed to protect bones and other estrogen sensitive tissue [18].

#### Ethynediol Diacetate

- Ethynediol is made from norethindrone and is easily converted to norethindrone within the body and therefor has the characteristics of norethindrone
  - ◦ Low progestational activity
  - ◦ Less androgenic than second-generation progestins but more than third and fourth generation progestins
    - ▪ Advantage: the androgenic activity in this progestin is beneficial when used in a combined oral contraceptive as it antagonizes estrogen’s stimulation of hepatic proteins [most importantly has some effect in reducing the increase in clotting factors caused by the estrogen in the COCP]
    - ▪ Disadvantage: No antiandrogenic activity [depends on estrogen in COCPs for reducing acne]
- Converted to estrogen: conversion rates of norethindrone and norethindrone acetate to ethinyl estradiol via aromatase [16, 17] see Fig. 1

### Second generation

Levonorgestrel and norgestrel

#### Levonorgestrel

- Levonorgestrel is the most widely prescribed birth control progestin worldwide
- Has strong progestational
  - ◦ Used in FDA-approved emergency contraception
- Has strong androgenic effects.
  - ◦ Advantage: the androgenic activity in this progestin is beneficial when used in a combined oral contraceptive as it antagonizes estrogen’s stimulation of hepatic proteins [most importantly has some effect in reducing the increase in clotting factors caused by the estrogen in the COCP]
    - ▪ Reported to have the lowest risk of blood clots compared to other combination birth control pills
  - ◦ Disadvantage: The androgenic activity increases the risks of side effects like acne, weight gain, and negative impact on cholesterol levels

#### Norgestrel

- Norgestrel Is a mixture of dextro-norgestrel and levonorgestrel [the active ingredient].
- Has high progestational activity
- Has strong androgenic effects
  - ◦ Advantage: the androgenic activity in this progestin is beneficial when used in a combined oral contraceptive as it antagonizes estrogen’s stimulation of hepatic proteins [most importantly has some effect in reducing the increase in clotting factors caused by the estrogen in the COCP]
  - ◦ Disadvantage: The androgenic activity increases the risks of side effects like acne, weight gain, and negative impact on cholesterol levels

### Third generation

Desogestrel and norgestimate

#### Desogestrel

- Has high progestational activity
- Has minimal to no androgenic activity [19]
  - ◦ Advantage: The lack of androgenic activity lowers the risk of side effects such as acne, weight gain, and other androgenic effects seen with first and especially second generation progestin COCPs.
    - ▪ Reducing menstrual cramps
    - ▪ Reduced risk of migraines
    - ▪ Positive effects on cholesterol
    - ▪ Less weight gain
  - ◦ Disadvantage: the lack of androgenic activity in this progestin is a real disadvantage when used in a combined oral contraceptive as it fails to antagonize the estrogenic stimulation of hepatic proteins [most importantly allowing for unchecked increases in clotting factors caused by the estrogen in the COCP]
    - ▪ Many reports of a higher risk of clotting with use of desogestrel COCPs, particularly with the higher dose estrogen COCPs.
      - Clinical trials show a possibly higher risk of non-fatal blood clots with desogestrel pills
      - Desogestrel in combination with 30 to 40 µg of Ethinyl estradiol is reported to have the highest blood clot risk of all combination birth control pills.

#### Norgestimate

- High progestational activity
- May has slight estrogenic effect
- Low androgenic impact [19]
  - ◦ Minimal effect on cholesterol and carbohydrate metabolism
  - ◦ May help to improve acne
    - ▪ Only FDA approved COCP approved to treating acne

## Fourth generation

Drospirenone

## Drospirenone

- Drospirenone is a new progestogen, derived from 17alpha-spirolactone
- The relationship between its progestogenic and its antimineralocorticoid potency is almost identical to that of natural progesterone [20].
  - ◦ Advantage: YAZ has FDA-approval to help treat the mood disorder premenstrual dysphoric disorder (PMDD).
  - ◦ Disadvantage: People with kidney, liver, or adrenal disease should not take pills containing this progestin as it may cause higher potassium levels.
  - ◦ It has low androgenic activity: Reportedly has antiandrogenic activity. [sc + ]
    - ▪ Cyproterone acetate (CPA) is the most potent antiandrogenic progestin, followed by dienogest, drosperinone, and chlormadinone acetate. Nomegestrol acetate and medrogestone also exert some antiandrogenic properties and are similar to chlormadinone acetate in antiandrogenic potency [21]
  - ◦ Advantage: The lack of androgenic activity lowers the risk of side effects such as acne, weight gain, and other androgenic effects seen with first and especially second generation progestin COCPs.
    - ▪ Reducing menstrual cramps
    - ▪ Reduced risk of migraines
    - ▪ Positive effects on cholesterol
    - ▪ Less weight gain
  - ◦ Disadvantage: the lack of androgenic activity in this progestin is a real disadvantage when used in a combined oral contraceptive as it fails to antagonize the estrogenic stimulation of hepatic proteins [most importantly allowing for unchecked increases in clotting factors caused by the estrogen in the COCP]
  - ◦ Drospirenone is linked to an increased risk of blood clots in several studies. A 2017 review looked at 17 studies on drospirenone and reported that risk of blood clots ranged from no increase to a 3.3 times increased risk compared to levonorgestrel, the birth control pill thought to have the lowest risk. [Schneid] the review concluded that the risk is only slightly increased.
  - ◦ In another study of 55,000 participants, the study found that the risk of blood clots was 3.19 times higher in COCP with drospirenone users than with COCP with levonorgestrel for first-time users. The risk was 1.96 times higher in restarters [11].
  - ▪ People who have other risk factors for blood clots may wish to consider a birth control pill other than those with drospirenone or desogestrel., or an entirely different form of birth control may be preferable.

## Summary

- The progestin revolution gained speed with introduction of a succession of LARC progestin-only contraceptive methods with excellent bleeding control. In women suffering from heavy bleeding, Mirena reduced the amount of bleeding by 80% after 3 months of use. After 6 months, bleeding was reduced by 90% [22]. Bleeding data for Liletta indicate that amenorrhea rates are about 40%, even into year six. Mirena reduced the amount of bleeding by 80% after 3 months of use. After 6 months, bleeding was reduced by 90%.
- A Cochrane review compared the efficacy of multiple combined hormonal contraceptives compared with other therapies in the treatment of heavy menstrual bleeding (HMB). While combined oral hormonal contraceptives were effective in reducing HMB, the levonorgestrel (LNG) releasing IUD was more effective [23].
- Fifty women planning on having surgery to treat their heavy periods agree instead to have Mirena inserted instead. Thirty-seven of the women reported that they noticed much lower amounts of blood loss after 3 months of Mirena use. This number increased to 41 after 9 months of use. Forty-one of these women decided to continue using Mirena instead of having surgery to treat their heavy bleeding [24].
- Six different research studies showed that when compared with endometrial ablation, Mirena was as effective in reducing monthly blood loss. Mirena was associated with fewer side effects and it did not affect future fertility [25].
- In a 1-year study in women with heavy bleeding, Mirena decreased blood loss in three out of four women —79.5% of the women planned to continue using Mirena. Hemoglobin levels increased at 3 and 12 months in women with the Mirena [26, 27].
- Mirena, hysterectomy, and endometrial ablation for heavy bleeding were compared. Mirena was ranked as best regarding the number of quality-of-life years, next was hysterectomy, followed by endometrial ablation [28].
- Progestins are increasingly becoming the treatments of choice to stop acute bleeding and/or control long-term bleeding. Progestin-containing IUDs are very effective for long-term bleeding control [28].
- While Depo-MPA can be associated with irregular bleeding, amenorrhea is an advantage to many users as 50% of users are amenorrheic at 6 year of use and 70% are amenorrheic after 2 years of use [29].
- Depo-MPA decreases dysmenorrhea, anemia and the risk of endometrial and ovarian cancer [30, 31].

## Conclusion

The progestin revolution has led to the design of a multitude of contraceptive options that offer a strong progestin component coupled with a limited or absent estrogen component. These products are designed to significantly decrease or abolish menstrual blood flow. Also linked to this ever-increasing appreciation of progestin’s strong role are advances in non-contraceptive protocols and products with improved bleeding control.including those using MPA oral pills with or without injectable MPA. The big “Plus” is the increasing appreciation of the variety of risks and benefit profiles and individual characteristics of the many progestins in the market today. This is opening doors to further development of products with added benefits such as acne treatment, eliminating weight gain and other androgen side effects, decreasing PMS, regimens that lower fluid retention, and products that can be used safely in older women or those with multiple cardiovascular risk factors.

The progestin revolution continues to grow.
